# Destabilizing Domains Mediate Reversible Transgene Expression in the Brain

**DOI:** 10.1371/journal.pone.0046269

**Published:** 2012-09-28

**Authors:** Khalid Tai, Luis Quintino, Christina Isaksson, Fredrik Gussing, Cecilia Lundberg

**Affiliations:** CNS Gene Therapy Unit, Wallenberg Neuroscience Center, Department of Experimental Medical Science, Lund University, Lund, Sweden; French National Centre for Scientific Research, France

## Abstract

Regulating transgene expression in vivo by delivering oral drugs has been a long-time goal for the gene therapy field. A novel gene regulating system based on targeted proteasomal degradation has been recently developed. The system is based on a destabilizing domain (DD) of the Escherichia coli dihydrofolate reductase (DHFR) that directs fused proteins to proteasomal destruction. Creating YFP proteins fused to destabilizing domains enabled TMP based induction of YFP expression in the brain, whereas omission of TMP resulted in loss of YFP expression. Moreover, induction of YFP expression was dose dependent and at higher TMP dosages, induced YFP reached levels comparable to expression of unregulated transgene., Transgene expression could be reversibly regulated using the DD system. Importantly, no adverse effects of TMP treatment or expression of DD-fusion proteins in the brain were observed. To show proof of concept that destabilizing domains derived from DHFR could be used with a biologically active molecule, DD were fused to GDNF, which is a potent neurotrophic factor of dopamine neurons. N-terminal placement of the DD resulted in TMP-regulated release of biologically active GDNF. Our findings suggest that TMP-regulated destabilizing domains can afford transgene regulation in the brain. The fact that GDNF could be regulated is very promising for developing future gene therapies (e.g. for Parkinson's disease) and should be further investigated.

## Introduction

The possibility to regulate transgene expression has been a discussed in the gene therapy field for a long time (see e.g. [1], [2]). In clinical settings, regulated transgene expression would allow for increased or decreased transgene levels in response to clinical need. Regulating transgene expression would ideally provide a means to avoid adverse effects due to continuous overexpression of therapeutic genes. Furthermore, the ability to turn transgene expression off and on offers experimental advantages when studying causal effects of gene transfer in disease models.

Many different regulated gene expression systems have been developed [3] and most operate at transcriptional levels. One of the most widely used inducible systems is based on tetracycline-responsive elements fused to strong activators or silencers (for a recent review see e.g. [4]). There is also a regulated transcriptional system based on FRAP and rapamycin that has been developed for use in gene transfer paradigms [5].

Gene therapy applications in the central nervous system represent a challenge for any gene regulation system developed so far, as the activating drug needs to cross the blood brain barrier to effectively mediate regulation of gene expression. When tested for gene transfer to the brain, most of the systems available so far have been significantly leaky [6]. Furthermore, long-term regulation and subsequent expression using current systems may be immunogenic and lead to decreased expression of the transgene over time. For example, tetracycline-regulated transgenes in the brain of monkeys have shown signs of immunogenicity [7]. Leakiness of existing systems combined with immunogenicity issues showed that there is a need for improved systems to effectively regulate gene expression.

Current gene regulation strategies have been further improved by using combination of activators and silencers [4], [8] or changing the building blocks for the transcriptional system, from tetracycline-responsive to rapamycin-responsive [9]. Moreover, mutations on the transcription factors can be created to make the systems more sensitive to induction, thereby increasing their operational window [4], [10]. However, these systems still fall short for in vivo therapeutic gene regulation in the brain.

Recently, Dr Wandless and co-workers developed a novel inducible system [11]. Instead of regulating transgenes at a transcriptional level, this new system directly regulates stability of the transgene product. The regulation is achieved by fusing the transgene product with a destabilizing domain, which renders the resulting fusion protein unstable and prone to proteasomal degradation. By adding a small molecule such as Shield-1, the protein is shielded from degradation and the transgene can be stably expressed. The Shield-1 inducible system, using FKBP as a DD has been shown to also be effective in vivo [12]. However, Shield-1 is a novel drug, its biodistribution is not fully characterized and it is not known to what extent Shield-1 crosses the blood-brain barrier.

Therefore, the Wandless group has recently developed another regulation system based on a destabilizing domain (DD) derived from Escherichia coli dihydrofolate reductase (DHFR), enabling the use of the small-molecule trimethoprim (TMP) as a stabilizer [13]. TMP is a well-characterized drug that crosses the blood-brain barrier and has been used safely as an antibiotic in humans both in therapeutic and long-term prophylactic regimes [14]. Using this novel DD variant, Iwamoto et al [13] has characterized in vitro kinetics and showed proof of principle induction of an YFP and DD fusion protein in rat brain by oral administration of TMP. Here, we further characterize the DD system in the brain by showing reversible regulation, in vivo dosage and kinetics of TMP regulation of YFP DD fusion protein expression. Furthermore, we show that the system has the potential to be applied to biologically active proteins since a regulated fusion protein of DD and glial cell derived neurotrophic factor (GDNF), a very relevant molecule for gene therapy in Parkinson's disease [15] can be constructed using this system and the resulting fusion protein is functional.

## Materials and Methods

### Lentiviral Vectors

**Figure 1:**
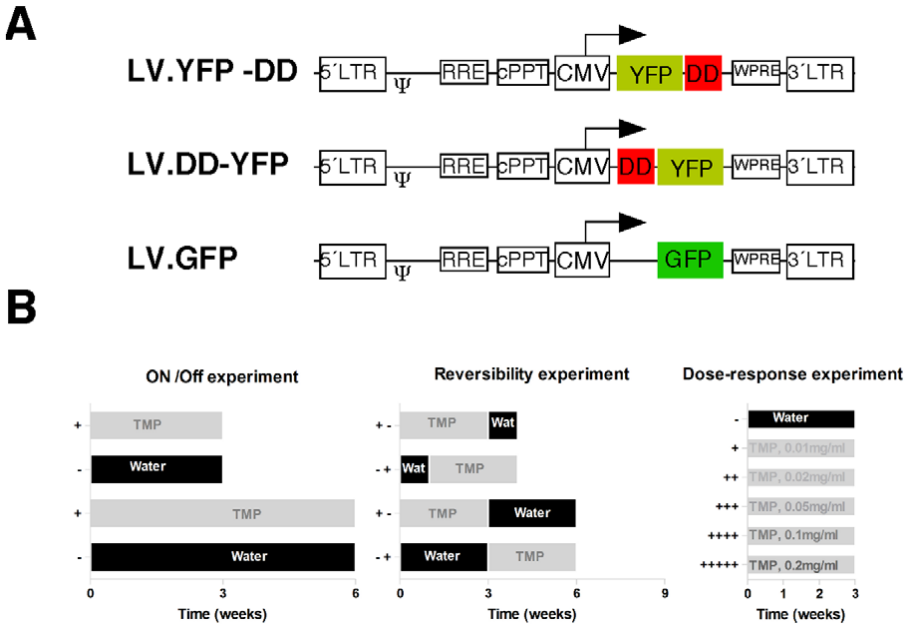
Vector details and experimental design. (A) A schematic view of the vectors used in the present study. All transgenes were expressed from the human CMV promoter, a central poly-purine tract (cPPT) was included as well as the post-transcriptional regulatory element WPRE. The destabilizing domain (DD) was placed either downstream or upstream of the yellow fluorescent protein (YFP). A vector expressing unregulated enhanced green fluorescent protein (GFP) was used as control. (B) Shows the different experimental designs. All groups included 5 animals.

Lentiviral vectors were constructed using Gateway® technology (Invitrogen). The human CMV promoter was placed upstream of the appropriate fusion gene in the lentiviral backbone 2k7neo [16] for the YFP constructs as described earlier (Figure 1a, [13]). The GDNF constructs were cloned into a modified lentiviral backbone, pBG, where the neo-cassette of the 2k7neo was removed by cutting with Kpn I and Age I, blunting the ends with Klenow DNA polymerase I and ligated using standard protocols. Both GDNF and YFP vectors carried the R12Y/Y100I-YFP or YFP-N18T/A19V versions for N-terminal and C-terminal fusion of the destabilization domains, respectively. The resulting vectors were named 2K7-CMV-YFP-N18T (C-terminal), 2K7-CMV-Y100I-YFP (N-terminal), pBG-CMV-GDNF-N18T (C-terminal) and pBG-CMV-Y100I-GDNF (N-terminal). All lentiviral vectors were produced and titered using quantitative PCR as described previously [17]. The titers of control lentiviral vectors expressing GFP used as reference in these experiments were estimated to be approximately 10^8^ TU/ml.

### Cell Culture

The cell lines TGW and 293T were cultured in DMEM containing 10% of fetal calf serum and penicillin/streptomycin. For the functional assay, 293T cells were transduced with an MOI of 2.5. At least 72 h after transduction, 4×10^5^ transduced 293T cells were seeded. On the next day, 3×10^5^ TGW cells were seeded and the 293T cells were treated with 2 ml culture media containing 1×10^−5^ M of TMP. Twenty-four hours after the TMP treatment, 293T cells and their respective media were harvested. One point eight ml of media was used to replace the original media of TGW cells and 24 hours after media replacement TGW cells were harvested. The remaining 200 µl of media was used to determine GDNF concentration by ELISA. The samples were diluted and the GDNF concentration estimated using the GDNF Emax Immunoassay ELISA (Promega) according to the manufacturers instructions.

### Cell harvesting and quantification for Western Blot

TGW and 293T cells were washed with PBS and incubated for 2 min in 60 µL and 200 µL lysis buffer (50 mM TRIS, pH 7.4, 150 mM NaCl, 1% Triton X-100) containing protease inhibitor cocktail (Complete protease inhibitor cocktail, Roche Applied Science) before being collected using a cell scraper. The lysed cells and buffer were incubated for 30 min at 4°C and then centrifuged for 10.000 g for 10 min at 4°C. The amount of protein in the supernatants was quantified using DC Protein assay (Bio-Rad) according to the manufacturers instructions.

### Western Blot

Samples containing 40 µg of protein were diluted 1∶1 in Laemmli Sample Buffer (Bio-Rad), incubated for 5 min at 99°C and placed immediately on ice. The samples were then loaded onto Criterion 10% Tris-HCl precast gels (Bio-Rad) and the separated proteins were transferred to an Immun-Blot PVDF Membrane (Bio-Rad) according to the manufacturers instructions. The transfer efficiency was analyzed using Ponceau solution. The membranes were washed three times in TTBS (0.9% NaCl, 20 mM Tris, pH 7.6, 0.1% Tween 20). Afterwards, the membranes were blocked in TTBS+5% milk powder for 1 hour at room temperature and washed three times in TTBS. The membranes were incubated in TTBS+5% milk powder containing primary antibody (anti tyrosine hydroxylase (TH), 1∶5000, Millipore) overnight at 4°C. The membranes were then washed for three times in TTBS and incubated in TTBS+5% milk powder containing secondary antibody (anti goat mouse- HRP, 1∶5000, Santa Cruz Biotechnology) for 1 hour at room temperature. After the incubation, the membranes were washed three times in TTBS, once in TBS and incubated in ECL plus (GE Healthcare) according to the manufacturers instructions. The presence of bands was analyzed in a Versadoc system (Bio-Rad). After the membranes were analyzed, the membrane was washed three times in TTBS and incubated in Stripping buffer (100 mM 2-mercaptoethanol, 2% SDS, 62.4 mM Tris-HCL pH 6.8) for 30 min at 50°C. The membrane was then rinsed in copious amounts of water, washed three times in TTBS and blocked in TTBS+5% milk powder for 1 hour at room temperature and washed a further three times in TTBS. The membranes were incubated in TTBS+5% milk powder containing primary antibody (anti β-actin-HRP, 1∶50000, Sigma). After the incubation, the membrane was washed and the presence of β-actin assayed as described above.

### Ethics Statement

All animals were cared for in accordance with the principles of the Guide to the Care and Use of Experimental Animals. All animal procedures were approved and performed according to the guidelines of the Ethical Committee for Use of Laboratory Animals at Lund University (#M9-10). Viral vector production and usage was approved by the Swedish work environment authority (#202100-3211 v61 and # 202100-3211 v36).

### Animals

Two months-old female Sprague–Dawley rats (Charles River) were used for the present study. Upon arrival, animals were quarantined for 5 days prior to any testing. Rats were housed with a 12∶12 hours light∶dark cycle with ad libitum access to food and water. Prior to the start of the study, animals were weighed and placed in experimental groups in a fashion that yielded equal average body weights among the groups. Trimethoprim (TMP, oral suspension 10 mg/ml, Meda AB, Sweden) was freshly administered in drinking water on a daily basis. Two days after lentiviral injections the animals were treated according to the experimental design (Fig. 1). The TMP dose was 0.1 mg/ml drinking water except in the dose response experiment where the dose ranged from 0.01–0.2 mg/ml. A total of 70 rats were used (n = 5 in each group). All animals were observed daily and weighed at weekly intervals.

### Surgical Procedures

Two microliter (corresponding to 4.3×10^7^–5.7×10^9^ TU) of lentiviral vectors (2K7-CMV-YFP-N18T, 2K7-CMV-Y100I-YFP) were injected bilaterally into rat striatum (left hemisphere and right hemisphere, respectively). A thin glass capillary was attached to a Hamilton syringe with a tube resulting in more precise injections with less brain damage. The stereotaxic injection coordinates were AP+0.5, ML ±3, and DV1 - 5, DV2 - 4 mm as measured from bregma.

### Immunohistochemistry

Animals were anesthetized using pentobarbital (250 mg/kg i.p., Apoteksbolaget) and perfused through the heart with ice-cold saline for 1 minute, followed by fixation with 4% paraformaldehyde in 0.l M phosphate buffered saline (pH 7.4) for 5 minutes at 50 ml/min. Rat brains were removed and suspended in 4% paraformaldehyde for 2 hours, then transferred to phosphate buffered saline containing 25% sucrose for 3 to 4 days at 4°C.

The brains were then sectioned on a freezing-stage microtome in 35 µm thick sections in a total of five series per brain. Primary antibodies used were rabbit anti-GFP (1∶20000, Abcam), mouse anti-NeuN (1∶100) (MAB377; Chemicon, Temecula, CA, USA) and mouse anti-CD11b (OX42) (1∶200)(MCA275G; Serotec, Raleigh, NC, USA). The secondary antibody for GFP bright field microscopy was biotinylated horse anti-mouse antibody (1∶200) (BA-2001; Vector Laboratories, Inc., Burlingame, CA, USA. Brain sections were incubated in ABC solution (Vectastain Elite ABC kit, Vector Laboratories) followed by development with 3,3-diaminobenzidine solution (DAB kit, Vector Laboratories) to visualize immunoreactivity. Brain sections were then mounted, dehydrated through ascending graded concentrations of alcohol, cleared in xylene and cover slipped using DPX mounting medium (BDH Chemicals, UK). Fluorescently labeled secondary antibodies were: Cy3 donkey anti-mouse (1∶400) (Millipore), Alexa488 goat anti-rabbit (1∶500) (Jackson Labs). After overnight incubation at 4°C with the primary antibody and 2 hours at room temperature with the secondary antibody, sections were rinsed with potassium-phosphate buffered saline (KPBS), mounted on coated slides and cover slipped with DABCO. Background controls where the primary antibody was omitted were performed to evaluate the unspecific staining by the procedures and were thus used to define what was immunopositive.

### Quantification

The total number of YFP positive cells counted in every 5th coronal striatal section was quantified in order to estimate the total number of YFP positive cells for each animal. The method of Abercrombie [25] was used to correct the double counting errors.

### Statistical Analysis

Statistical analyses were performed using one-way ANOVA, followed by Bonferroni *post hoc* test for comparison with the corresponding control groups. *P* values<0.05 were considered significant.

## Results

The first experiments were performed to evaluate to what extent TMP could stabilize YFP fused to a DD at the N- (Y100I; DD-YFP) or C-terminal (N18T; YFP-DD) *in vivo*. Rats received bilateral striatal injections of lentiviral vectors (Lv) Lv.YFP-DD and Lv.DD-YFP (left and right hemisphere, respectively) and were then allocated into TMP treatment or water only groups. As a positive control we used injections of Lv.GFP in a separated set of animals (Figure 1a). Two days after vector injection into the striatum, experimental groups received a dose of TMP (0.1 mg/ml) in the drinking water, as outlined in Figure 1b, and the body weight was measured weekly. There was no difference in the body weight or water intake between any of the groups (data not shown), indicating that the TMP regimen was well tolerated by the animals.

**Figure 2:**
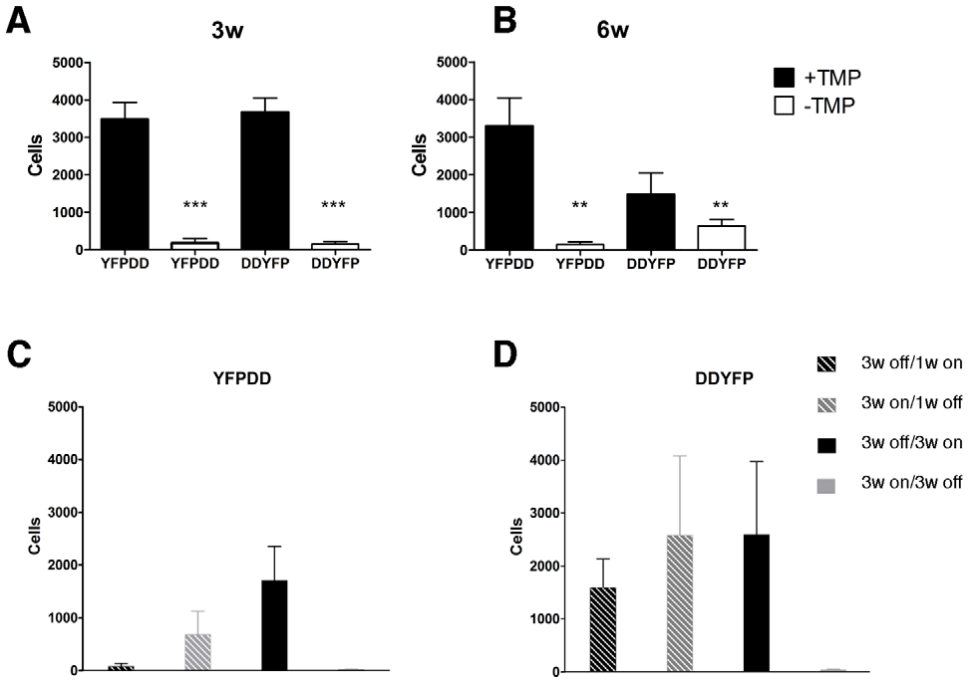
Regulation of YFP by destabilizing domains in vivo. Histograms showing the number of YFP expressing cells in the transduced striatum at 3 weeks (A) and 6 weeks (B) post injections. Addition of 0.1 mg/ml TMP to the drinking water induced expression of transgene expressing cells in all animals. The effect was not dependent on whether the DD was fused to the N- or C- terminus of YFP. (C) and (D) show the dynamics of YFP protein expression 1 week or 3 weeks with or without TMP, for YFP-DD and DD-YFP, respectively. One week of adding TMP to the drinking water or omitting it was not enough to fully regulate the protein expression ,On the other hand, 3 weeks of treatment was sufficient to reach levels of YFP expression similar to those attained from chronic TMP treatment or no TMP at all, respectively. *** = *p*<0.001, ** = *p*<0.01.

**Figure 3:**
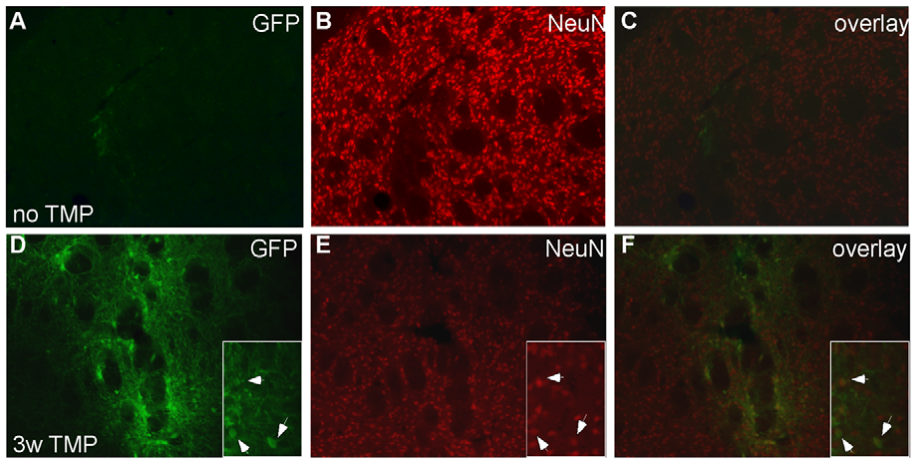
Immunofluorescence for GFP and NeuN. A–C shows the results form an animal that did not receive any TMP in the drinking water. D–F shows the results from an animal that received 0.1 mg/ml TMP in the drinking water for 3 weeks. Note the large number of GFP positive cells in the injected striatum. The squares at the bottom right show enlargements of colabeled cells, arrows indicate examples. A,D: GFP staining; B,E; NeuN staining; C, F: overlay.

At 3 weeks, Lv.DD-YFP yielded 3672±378 YFP immunopositive cells (as determined by a rabbit- anti GFP antibody staining) and the Lv.YFP-DD injections resulted in 3478±453 YFP positive cells (Figure 2a, Figure 3). This expression level was in contrast to the scattered cells detected throughout striata of control animals that drank only water throughout the experiment (149±67 and 180±113 cells in DD-YFP and YFP-DD groups, respectively; Figure 2a, Figure 3).

Similarly, at six weeks of continuous TMP treatment, significant numbers of YFP expressing cells could only be detected in the striata of animals treated with TMP in the drinking water (3297±746 and 1474±573 positive cells in YFP-DD and DD-YFP, respectively, Figure 2b).

At no time point did we detect any signs of pathology as measured by light microscopy or immunostaining for CD11b (data not shown), a common microglial marker known to be upregulated during neuroinflammation (see e.g. Jiang et al, 2009). Double staining with a GFP antibody (that cross reacts with YFP) and the neuronal marker NeuN revealed that the vast majority (90±4%) of YFP expressing cells were NeuN positive, indicating that the transduced cells were neurons.

### DD regulation is reversible in vivo

In order to verify the hypothesis that DD system provides a reversible control of YFP expression *in vivo*, we treated rats first with normal drinking water and then with TMP, and vice versa (Figure 1b). We used two different times, 1 week and 3 weeks, to evaluate the ability of the DD system to turn transgene expression off and on. We found that 1 week of TMP treatment was not sufficient to induce a robust YFP expression (80±58 and 1593±540 positive cells in YFP-DD and DD-YFP, respectively; Figure 2c–d) nor was one week without TMP sufficient to fully destabilize the YFP to background levels (686±444 and 2585±1494 positive cells in YFP-DD and DD-YFP, respectively; Figure 2c–d)

The data indicates that the DD-regulated vector system allowed an efficient regulation of transgene expression *in vivo* when the rats were treated for 3 weeks with water followed by 3 weeks with TMP (2591±1378 and 1708±649 positive cells in YFP-DD and DD-YFP, respectively; Figure 2c–d). In addition, 3 weeks of TMP treatment followed by 3 weeks of water treatment turned off expression of the YFP-DD fusion proteins, demonstrating that TMP protection of DD is reversible in vivo (23±7 and 38±15 positive cells in YFP-DD and DD-YFP, respectively; Figure 2c–d).

### YFP-DD expression is stabilized in a dose dependent manner

**Figure 4:**
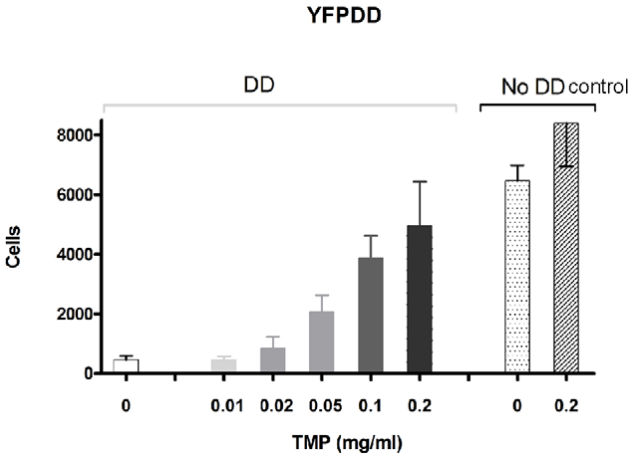
Dose-response histogram for YFP regulation. The number of YFP expressing cells in the striatum increases in correlation to increasing TMP doses. The number of GFP expressing cells from a non-regulated GFP control vector is also included in the histogram. There were no significant differences between the numbers of GFP expressing cells in the 0.1 and 02 mg/ml TMP groups compared to the control Lv.

Next we set out to investigate if stabilization of YFP-DD in the rat striatum was dependent on TMP dosage. The animals were injected with 2 µl of vector in the striatum and two days later they were given TMP at a dose range of 0.01–0.2 mg/ml in the drinking water (Figure 1b). Three weeks later the animals were sacrificed and the number of YFP expressing cells was estimated. Again, TMP had no effect on body weight or water intake (data not shown) nor did it affect transgene expression of the control vector Lv.GFP (Figure 4). Moreover, we did not detect any statistically significant differences in the number of positive cells in YFP-DD group receiving the highest TMP dose and control vector Lv.GFP with or without TMP (4962±1483 YFP-DD 0.2 mg/ml TMP vs. 8398±1447 GFP 0.2 mg/ml TMP and 6465±521 no TMP, Figure 4).

### Fusion proteins of GDNF and DD are regulated and functional in vitro

**Figure 5:**
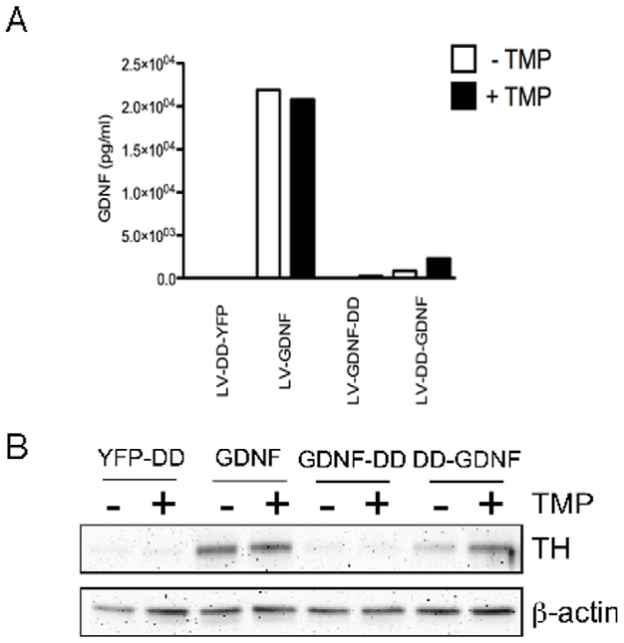
Regulating GDNF expression using DD. (A) Histogram showing the levels of GDNF produced from DD-YFP vector, wild type GDNF, GDNF-DD and DD-GDNF, as measured by GDNF ELISA. C-terminal fusion of the DD to GDNF did not result in any GDNF production, while N-terminal DD-GDNF fusion resulted in TMP-induced GDNF production. Furthermore, the DD-GDNF was functional in that it induced tyrosine hydroxylase expression in the TGW cell line as shown in (B).

To evaluate to what extent DD technology can be generalized and used for a biologically active protein, fusion proteins of the neurotrophic factor GDNF and DD were designed. GDNF is a very potent neurotrophic factor for dopamine neurons in the substantia nigra [15] and thus a possible future therapeutic protein. To show proof of concept, lentiviral vectors expressing GDNF and DD fusion proteins transduced 293T cells and ELISA was used to determine GDNF secretion. The C-terminal design (GDNF-DD) had minimal expression levels upon induction. On the other hand, N-terminal placement of DD (DD-GDNF) allowed an increase in secretion of GDNF in a TMP dependent manner. Addition of TMP to the culture media resulted in a 2.7 fold induction of DD-GDNF secretion into the media. The level of GDNF from the N-terminal design was 11% of wild type (2.3 ng/ml vs. 20.8 ng/ml respectively, Figure 5) in the induced state.

The next step was to ensure that the GDNF product resulting from DD induction was biologically active. To test this, conditioned media from the transduced 293T cells, with or without TMP induction was added to the GDNF responsive cell line TGW [18]. The TGW cells endogenously express rearranged during transfection (RET) and GDNF family receptor alpha 1, which are the canonical receptors for GDNF. It has been previously shown that treatment of TGW cells with GDNF activates signaling cascades that result in robust and rapid upregulation TH expression. Therefore, TGW cells provide a suitable reporter cell line for monitoring GDNF activity. Treatment of TGW cells with media from 293T cells transduced with DD-GDNF and induced with TMP resulted in an upregulation of TH that was detected by Western Blot (Figure 5). Wild type GDNF induced a robust TH expression as well, while GDNF-DD failed to have any effect on TH upregulation. These results suggested that DD technology could be used to regulate biologically active proteins with therapeutic potential, such as GDNF.

## Discussion

In the present study we evaluated the feasibility of using destabilizing domains fused to transgenes to regulate levels of transgene product. Using YFP as a reporter, we showed that TMP could be readily used to induce expression in the brain in a dose-dependent manner and without detectable side effects. . We further tested the application of the system to biologically active proteins by fusing DD to neurotrophic factor GDNF and showed that we generated regulated and biologically active protein.

The addition of TMP to drinking water stabilized the DD-fusion proteins and induced YFP expression in the rat striatum. These findings are in line with and extend our previous observations [13]. The expression level after induction was in the same range as control vectors overexpressing unregulated GFP, indicating that TMP was able to stabilize the DD-fusions in the vast majority of transduced cells in the striatum. Furthermore, the widespread transgene expression caused by TMP induction suggests that the DD-regulated transgene expression could be therapeutically relevant. Lentiviral vectors with gene expression levels similar to the controls used in our study have been effective in animal models of Parkinson's disease [6], [19], [20].

In control groups, 3 and 6 weeks of water treatment completely destabilized YFP DD fusion proteins, demonstrating that the DD approach is suitable for regulating protein stability in vivo. This is in accordance with the results of an alternative DD system that enabled a stringent regulation of YFP expression in mammalian cells treated with shield1, a synthetic ligand [11]. Moreover, the results obtained in our study are comparable to efficient regulation of GFP expression reported in vivo using classical transcriptional regulatory systems [19], [20].

The expression leakage in off state was small, about 4% of induced levels at 3 weeks, which is considerably less than using doxycycline system in rat model [6]. Nevertheless, the biological extent of this level of leakage will be addressed in subsequent studies.

In the present study we found that 3 weeks of TMP was needed to fully protect the DD-fusion proteins from degradation, while 1 week was not sufficient. Similarly, 1 week of washout was not enough to reach non-induced baseline transgene levels. On the other hand, 3 weeks after stopping TMP treatment were sufficient to loose YFP staining, indicating that the system is fully reversible in vivo. The observation that DHFR DD regulation can be reversed in vivo is in agreement with the reversibility of FKBP destabilizing domains described in a recent study from the Wandless group [12]. Moreover, giving increasing doses of TMP to the animals revealed a dose-dependent stabilization of the YFP-DD, in line to what has been shown using the FKBP based destabilizing domain system [12]. These results imply that DD-fusion of therapeutic proteins could be used and their effects could be adjusted by the dosage of the inducing drug.

The in vivo kinetics of YFP induction are however, slower when compared to in vitro regulation where changes in transgene levels can be detected within minutes from TMP addition to cell culture [13]. The observed results are most likely due to the pharmacokinetics of oral TMP administration. A prerequisite for a compound to cross the blood-brain barrier is to be lipid-soluble, which also results in an accumulation in body fat if the compound is given chronically (as in the present study). As a consequence, dynamics for achieving adequate plasma levels and washout are inherently slow [21]. This fact should be taken into consideration when designing translational studies using this system. For diseases that take years to develop, such as neurodegenerative disease, the slow kinetics may not be a disadvantage. Rather it may be an advantage ensuring a more stable transgene expression with less variation in TMP plasma levels.

To screen for possible adverse effects of TMP treatment, we measured water consumption and body weight of the animals treated TMP compared to water treated rats and found no differences between control and treated animals. Furthermore, we did not detect any difference in behavior such as ambulation, grooming, drinking, and eating. Considering that the dose was 0.01–0.2 mg/ml of TMP in the drinking water of animals, that on average rats drink 20–30 ml of water/day and that the animals weighted 250–300 g, we estimate that the dosages used in the study were between 0.8–20 mg/kg/day. As the dosage in humans is 8–20 mg/kg/day [22], we estimate that the dosages used in our study comparable to the standard dosages in humans. Furthermore, TMP is safe for chronic oral administration at the dosages used in our study, which is supported the fact that TMP is used as a prophylactic drug without adverse events in children receiving treatment for acute lymphoblastic leukemia [23] as well as in patients going through total knee arthroplasty [24].

When developing novel systems for gene delivery is in vital to assess side effects at the delivery site. In addition to analyzing the overall morphology of the transduced tissue by light microscopy, we investigated possible pathology using a standard marker of inflammation, namely microglial activation measured by CD11b immunostaining. We did not detect any difference compared to striata injected with wild type unregulated GFP. The lack of inflammation was comparable to previous findings using this Lv [17]. Moreover, we investigated the phenotype of the cells expressing the YFP and DD fusions and determined that 90% where neuronal at all analyzed time-points. The CMV promoter used here is activated by inflammation and has been shown to be more active in glia during inflammation [17], [25]. Thus, any inflammation caused by the DD system would lead to increased transgene product that would further potentiate the inflammation. Therefore, our finding further strengthens the interpretation that the DD-YFP fusion proteins did not induce any detectable inflammation in the striatum.

The DHFR DD used in our study showed reversible and dose-dependent expression of YFP in the striatum of animals. Furthermore, our study indicates that the DD system can be considered a viable alternative to currently inducible systems based on transcriptional regulation of gene expression for transgene regulation in the brain.

In this study we also wanted to prove that the DD technology could be applied to a biologically active protein with therapeutic potential and turned our initial efforts to GDNF, a potent trophic factor for dopamine neurons in the substantia nigra [15]. In a proof of principle experiment, we could show that GDNF resulting from DD regulation was released, regulated by TMP and functional in a bioassay when the DD was fused to the N-terminus. Although the levels of induced DD GDNF are lower than unregulated GDNF expression, DD GDNF was still able to be biologically active. Thus, it is possible to regulate a secreted, biologically active DD fusion protein using TMP. We are currently optimizing DD GDNF regulation and induction for in vivo studies. This finding leaves us very optimistic for future development of this system for use in gene therapy in general, and, taken into account the bioavailability of TMP in the brain, for gene therapy to the CNS in particular.
